# Flipped classroom in EFL: a teaching experience with pre-service teachers

**DOI:** 10.3389/fpsyg.2023.1269981

**Published:** 2023-11-17

**Authors:** Lenka Birova, Raúl Ruiz-Cecilia, Juan Ramón Guijarro-Ojeda

**Affiliations:** Department of Language and Literature Teaching, University of Granada, Granada, Spain

**Keywords:** flipped classroom, English language teaching, blended learning, listening skills, grammar skills

## Abstract

This research aims to test a flipped classroom model to improve students’ English proficiency. To achieve this goal, two research questions were posed: RQ1 “Does the suggested model of flipped classroom teaching strategy increase the learners’ accuracy in the use of grammar in the target language more than the non-flipped active-learning strategy used?” and RQ2 “Does the suggested model of Flipped Classroom teaching strategy increase learners’ listening skills in the target language more than the non-flipped active-learning strategy used?” The participants involved in the study were 55 students from the Faculty of Education, University of Trnava (Slovakia), comprising 45 females and 10 males. All participants were pre-service teachers of English language and literature in their first year of undergraduate studies. The research had a semi-experimental pre-test/post-test design which was given to the control and the experimental group. The results show that students in the flipped classroom had a statistically significant positive effect on the participants’ listening skills. As for grammar, both the control and the research group improved, but the results were not statistically significant. These findings partially match former studies, where language accuracy was also an indicator of flipped classroom success. The implications of this research are high since listening, often referred to as the “Cinderella” of language skills, has frequently been overlooked in EFL classes, leading to students not reaching expected proficiency levels.

## 1 Introduction

The flipped classroom (Bergmann and Sams, 2012) is a modern teaching strategy based on exchanging the content traditionally delivered through lectures with material typically assigned as homework. This teaching approach is suitable for learners of all ages, mastery levels, and formal education tiers. It can be applied to a wide range of courses and subjects. If implemented effectively, it has the potential to significantly transform the formal learning experience for all stakeholders involved. This transformation can lead not only to improved academic achievement but also to a higher subjective feeling of satisfaction among participants. The concept of the flipped classroom (also known as flipped learning or flipped instruction) is not entirely new. It builds upon principles from various instructional modes, spanning from ancient to modern times, and allows for adaptations by practitioners based on their specific needs. Notable influences on the flipped classroom include Khan Academy and other Massive Online Open Course providers, blended learning, Just-In-Time Teaching methodology, Bloom’s Taxonomy (Bloom et al., 1984), and others.

The flipped classroom is not officially codified. There is no single proper mode of its application, nor a specified set of rules to be followed in order to apply it. Due to this, the flipped classroom may seem difficult to define and, consequently, apply or examine. However, in its modern form as described here, this teaching strategy encompasses several key characteristics and principles that distinguish it as flipped. These include a focus on student activity during class, a preference for tasks that emphasize higher-order thinking skills, a departure from traditional teacher-student roles, the fostering and support of learner autonomy, a self-directed approach to learning, and learner responsibility, among others (Demirel, 2016). While the original proponents of the flipped classroom did not consider the use of technology necessary, in this work, we envisage it as one of the pillars of the strategy. This is to reflect the realities of the 21st-century world we live in and align with the goals of 21st-century education (OECD, 2019). Despite the existence of teaching strategies and methods with certain features similar or even identical to those of the flipped classroom, as well as older hobbyist, professional, and scientific publications, the true birth of the flipped classroom as we understand it today began in recent years. It was the activities of Bergmann and Sams (2012) who published their flipped lessons online and made them freely accessible that quickly garnered attention not only from their own students but also from students and teachers beyond their immediate community. Moreover, their book *Flip Your Classroom: Reach Every Student in Every Class Every Day* (2012) became a catalyst for the flipped classroom to evolve into a movement and the term itself to become a buzzword among professionals worldwide.

With the growing popularity of the flipped classroom, the amount of published research has naturally increased in recent years at an exponential rate. Around the year 2012, only a few dozen publications existed. However, as of the second quarter of 2023, a simple search of the term “flipped classroom” on the web search engine Google Scholar yields an incredible 287,000 articles and publications on the flipped classroom model itself or associated topics. The search engine lists publications specifically focused on the flipped classroom teaching strategy in a variety of teaching and learning settings from all over the world, spanning at least the first 98 pages of results. Not only has the volume of publications sharply increased, but the quality of the papers, variety of research methods and questions, and researcher-specific adaptations of the flipped classroom strategy have also improved. In the early years of the flipped classroom, the majority of publications were how-to guides written by enthusiastic teachers, which often lacked actual research aims or outcomes. It took some time for the innovative strategy to catch the attention of researchers studying foreign language learning and teaching, especially with regard to the English language. Initially, the first papers published on the uses and effectiveness of the flipped classroom model were primarily related to medical and pharmaceutical science university programs. In these programs, instructors appreciated the benefits of moving theoretical lectures to individual study time and dedicating class time to practical application. Natural sciences, in general, have shown particular adaptability to the flipped classroom approach. Even the pioneering duo of Bergmann and Sams (2012) started experimenting with pre-class video lectures in their chemistry lessons. However, teachers and researchers focused on other subjects quickly caught up, and the flipped classroom is now being implemented across a wide spectrum of subjects, learning programs, age groups, and learning cultures. In the early days, most of the initial research was conducted in the United States, which is understandable considering Bergmann and Sams’s (2012) role in starting the flipped movement there. However, since then, a considerable amount of research has emerged from all over the globe, with the Middle East and East Asia being particularly productive in recent years. There are still relatively few publications or knowledge about the topic in Central Europe, possibly due to the older generation of educators, who are often prominent among university faculties, having less proficiency in the English language, in which the majority of research on the flipped classroom is published.

It should be noted that the flipped classroom is not universally praised, and as its notoriety increases, criticism of this teaching approach is also growing. Some skeptics question the importance of the theoretical lecture and argue that the perceived success of the flipped classroom is not necessarily connected to the flip itself (Jensen et al., 2015). Insufficient consideration for the issues of digital-divide (Centeio, 2017), increased screen time for learners (Skooler, 2018), and the time investment required from teachers are among the points raised by dissenting reviewers. These critics caution against viewing flipped teaching as a universal solution to the numerous challenges in education today. All of the mentioned concerns, and more, deserve consideration when evaluating the effectiveness of flipped teaching in one’s own classroom. Nonetheless, it can be argued that the flipped classroom represents a valid attempt to align teaching and learning with the needs of the 21st century. It can be seen as a compromise between traditional and alternative education, bridging the gap between the old and the new, and one that may be acceptable and adaptable for both sides.

The flipped classroom is an umbrella term that encompasses a wide range of specific teaching approaches, ultimately depending on the individual teacher’s preferred style. Despite this variability, it is possible to identify a set of principles that most adaptations of flipped lessons adhere to Lara-Freire and Rojas-Yumisaca (2022). These include the reversal of lecture and homework content, a focus on higher-order cognitive skills during in-class activities, learner-centeredness and learner activity, a deviation from traditional teacher and learner roles with an emphasis on learner autonomy, and the use of modern technology for educational purposes. From a practical standpoint, the flip of lecture and homework content, which is traditionally seen as the defining characteristic of this teaching strategy, remains the most obvious and central aspect.

The process typically follows the following steps (Bergmann and Sams, 2012):

1. The teacher records their lecture or selects one from available sources and shares it with the students, often by posting it online. The lecture contains all the necessary information for students to actively participate in the class.
2. Students engage with the lecture independently as homework before attending the corresponding in-class session.
3. During class time, practical activities based on the lecture information are conducted. The teacher provides guidance to the students as needed, but the majority of the activity is carried out by the students themselves.

The range of in-class activities can include projects, exercises, writing assignments, and other tasks typically assigned for individual engagement as homework. Additionally, activities that promote a deeper understanding of the topic and encourage higher-order cognitive skills are incorporated. The flipping of content serves a deeper purpose. The rationale behind it is that while learners can passively receive information on their own, they may struggle to complete practical assignments without further guidance from the instructor. If students fail to fully comprehend the information provided in class, whether due to lack of attention, absence, or incomplete understanding, their ability to successfully complete assignments based on that information is hindered. In the best case, they seek external help but may still fail to complete the assignment correctly. In the worst case, they may not complete it at all. This diminishes the assignment usefulness, fails to fulfill its purpose, and hinders the learning goal. Moreover, the inability to complete assigned tasks can decrease enthusiasm for the subject, motivation for learning, and lead to feelings of self-doubt and lower self-esteem, which further complicates the learning process both in the present and the future.

In a traditional non-flipped setting, particularly for younger students, parents may often feel compelled to assist their children with homework assignments, even when they lack the necessary competence to do so. This frequent need for outside help can lead to frustration. Foreign language learners, due to the inherent complexity of the subject, are especially susceptible to requiring external assistance if they struggle to grasp the content taught in class. Without access to guidance from someone proficient in the target language, these students can encounter significant challenges when completing assignments outside of class.

To address this issue, the flipped classroom model offers a solution. By making instructors available during practical task sessions, these problems can be effectively mitigated. Teachers play a vital role in ensuring that tasks are executed correctly, providing guidance and support when needed, offering motivation during challenging times, and delivering real-time feedback to facilitate learning. Numerous studies have explored the positive impacts of the flipped classroom approach on foreign language learners. Abdullah et al. (2020) conducted pioneering research on the effects of the flipped classroom on learners’ self-confidence in using the English language. Umutlu and Akpinar (2020) provided evidence of the impact of different video modalities on writing achievement in flipped English classes. Yang and Chen (2020) focused their research on the uses of the flipped classroom in EFL, and although the differences between the control and research groups were not significant, both teachers and students recognized the potential of this approach. Other studies, such as the one developed by Namaziandost et al. (2020) or Namaziandost and Çakmak (2020), have measured students’ self-efficacy in EFL, concluding that the flipped classroom has a significant positive effect. Furthermore, the combination of the flipped classroom and gamification in the language classroom has been shown to improve students’ self-confidence and their ability to engage in self-directed learning (Birová, 2019; Zou, 2020).

The main objective of this research is to test a flipped classroom model to improve students’ English proficiency.

This study will attempt to find answers to the following research questions:

*RQ1*. Does the suggested model of Flipped Classroom teaching strategy increase the learners’ accuracy in the use of grammar in the target language more than the non-flipped active-learning strategy used?

*RQ2*. Does the suggested model of Flipped Classroom teaching strategy increase learners’ listening skills in the target language more than the non-flipped active-learning strategy used?

## 2 Materials and methods

### 2.1 Context

The experiment was conducted over a period of 7 weeks during the Summer Semester at the Department of English Language and Literature, University of Trnava (Slovakia). It took place within the course *Communication Language Skills 2*, which is a compulsory undergraduate program for Teaching English and Literature students.

### 2.2 Participants

The participants involved in the study were 55 students (45 females and 10 males) from the Faculty of Education. All participants were pre-service teachers of English language and literature in their first year of undergraduate studies. Among the participants, 19 were studying to become teachers of English language only, while 36 were enrolled in two-subject teaching programs. With the exception of one participant, all were citizens of Slovakia and Slovak was their mother tongue. Three students listed multiple mother tongues, including German, Russian, and Ukrainian. The age of the students ranged from 18 to 24 years, with an average age of 20. Prior to attending university, the students had studied English for a period of 7 to 18 years. In addition to studying English as part of the compulsory state curriculum for an average of 13 years (17 students) or 11 years (10 students), 27 students indicated that they had taken extra classes in English language for an average of 2.5 years. Furthermore, 33 students reported proficiency in another foreign language besides English. On the other hand, more than half of the students had not traveled to an English-speaking country, with only 17 students having spent more than 7 days in one. Additionally, 39 students were the first generation in their family to attend university, and the parents of 35 students did not speak English. However, 37 students reported that their parents spoke a foreign language, predominantly Russian (30). Many students had daily exposure to English outside of school through activities such as listening to music in English (45), watching videos in English (34), using educational applications (28), or reading (20). Notwithstanding, 25 students did not use English for personal communication outside of school.

In all groups, the researchers assumed the role of instructors throughout the duration of the experiment. Two colleagues from the faculty of the department where the experiment was conducted, who would later take over as instructors for two of the groups (one in the intervention group and one in the control group), visited the lessons and conducted class observations.

### 2.3 Ethics statement

Prior to the start of the experiment, informed consent was obtained from all participating students. However, there were two students in the same group who did not provide consent. While their outcomes were not considered in the results of the experiment, they were still involved in all the proceedings as part of their study group.

It is important to note that obtaining informed consent and respecting the decisions of students who did not provide consent is essential for ethical research practices. After getting informed consent, a pre-test was administered to all participants to assess their initial level of English language proficiency. The same exam was given to all groups involved in the study.

### 2.4 Data collection

The study employed a quantitative approach. A pre-test/post-test design was implemented to assess the effectiveness of the Flipped Classroom model in improving participants’ English language proficiency and to compare it with the traditional method. While the exams used in pre/post-test were different, they were equal in terms of focus, design, and types of exercises included. The tests consisted of a section assessing listening comprehension and another section testing the students’ proficiency in grammar, vocabulary, and text comprehension. The exam tasks were selected from the validated tests *Oxford Placement Test 1 and 2* (Allan, 2004a) and the *B2 First proficiency exam* (Cambridge Assessment English, 2015).

### 2.5 Data analysis

In the data analysis process, the statistical software SPSS (IBM, v26.0.0.1) was utilized to analyze the data collected, which was initially compiled using Google Sheets. As mentioned earlier, the participants were divided into a test group and a control group, consisting of 36 and 19 participants, respectively. To address any initial variability between the groups, an analysis of covariance (ANCOVA) was employed. The pre-test scores were used as covariates to control for any differences between the groups. The ANCOVA analysis was conducted following the instructions provided on the Laerd Statistics webpage. Each time the analysis was performed, the eight assumptions of the test were checked, adhering to the suggestions outlined in the steps to conducting a one-way ANCOVA on Laerd Statistics’ platform (Laerd Statistics, 2018).

The results of these tests are suppressed throughout for the sake of clarity but have been conducted, and the criteria met, in all cases.

The assumptions checked for ANCOVA analysis were as follows:

1. The dependent variable and covariates were measured using continuous scales.
2. The independent variable consisted of at least two categorical, independent groups (control and test groups).
3. The observations were independent of each other.
4. Significant outliers, if present, were removed from the analysis.
5. The residuals were tested for approximate normal distribution using the Shapiro–Wilk test of normality in SPSS.
6. Homogeneity of variances was assessed using Levene’s test for homogeneity of variances in SPSS.
7. The covariate was examined for linear relationship with the dependent variable at each level of the independent variable, which was established by plotting a grouped scatterplot of the covariate, post-test scores of the dependent variable, and independent variable in SPSS.
8. Homoscedasticity was checked by plotting a scatterplot of the standardized deviations of the residuals against the standardized predicted values.

### 2.6 Process

The study design was semi-experimental due to the limitations of group allocation. The students’ group assignments were predetermined based on their study program and their choice of a specific study group in the schedule before the experiment began. The already formed study groups were then designated as either experimental or control groups, with two groups in each category. However, shortly after the experiment started, the two smallest study groups, one of which had received the intervention and the other had not, were merged into one group based on a decision by the administrative body of the department. This combined group continued as an intervention-receiving group.

The experimental groups were provided with pre-class materials in the form of videos and video-lectures, which were shared with them through the online-based educational platform Rcampus. These audio-visual materials were sourced from YouTube, as it was determined that the quality of the available content was sufficient for the experiment purposes, eliminating the need to create authorized materials. This decision was also made to reduce the instructors’ workload in lesson preparation. Typically, each lesson had a video-lecture focusing on grammar and a video introducing the topic. The combined duration of the two videos was a maximum of 12 min, with most being around 10 min. The experimental groups were assigned tasks related to each topic video, which they were expected to complete before attending the corresponding class. These assignments were then used for analytical, evaluative, and synthetic tasks during in-class activities such as group discussions, paragraph writing, and chart creation. The video-lectures provided theoretical explanations of grammatical points, allowing more time for interactive and communicative tasks during face-to-face lessons. The control group did not receive the videos; instead, grammar instruction was conducted in-class, and topics were covered using textbook activities supplemented with instructor-created materials. In all groups, the in-class activities during the experiment were a combination of textbook exercises and materials developed by the instructors. These activities were chosen based on the principles of active learning and a student-centered approach, with a focus on communication and oral interaction. Pair work and group work were frequently utilized, and tasks emphasized higher-order thinking skills according to the revised Bloom’s Taxonomy (Anderson et al., 2001). Speaking and listening tasks were the most commonly used, while written production tasks were included in every lesson to a lesser extent. Reading exercises and drills were assigned as voluntary individual homework tasks, accessible through the virtual classroom on Rcampus. All groups followed the curriculum outlined in the *English File: Upper-intermediate Student’s Book* (3rd edition) by Latham-Koenig and Oxenden (2014), which served as the official textbook for the course.

Following the pre-test, the main part of the experiment, which involved the intervention, was conducted exclusively with the experimental group(s) over a period of 5 weeks. The post-test aimed to measure any changes in the participants’ language proficiency after the intervention.

## 3 Results

### 3.1 Listening comprehension

**Figure 1:**
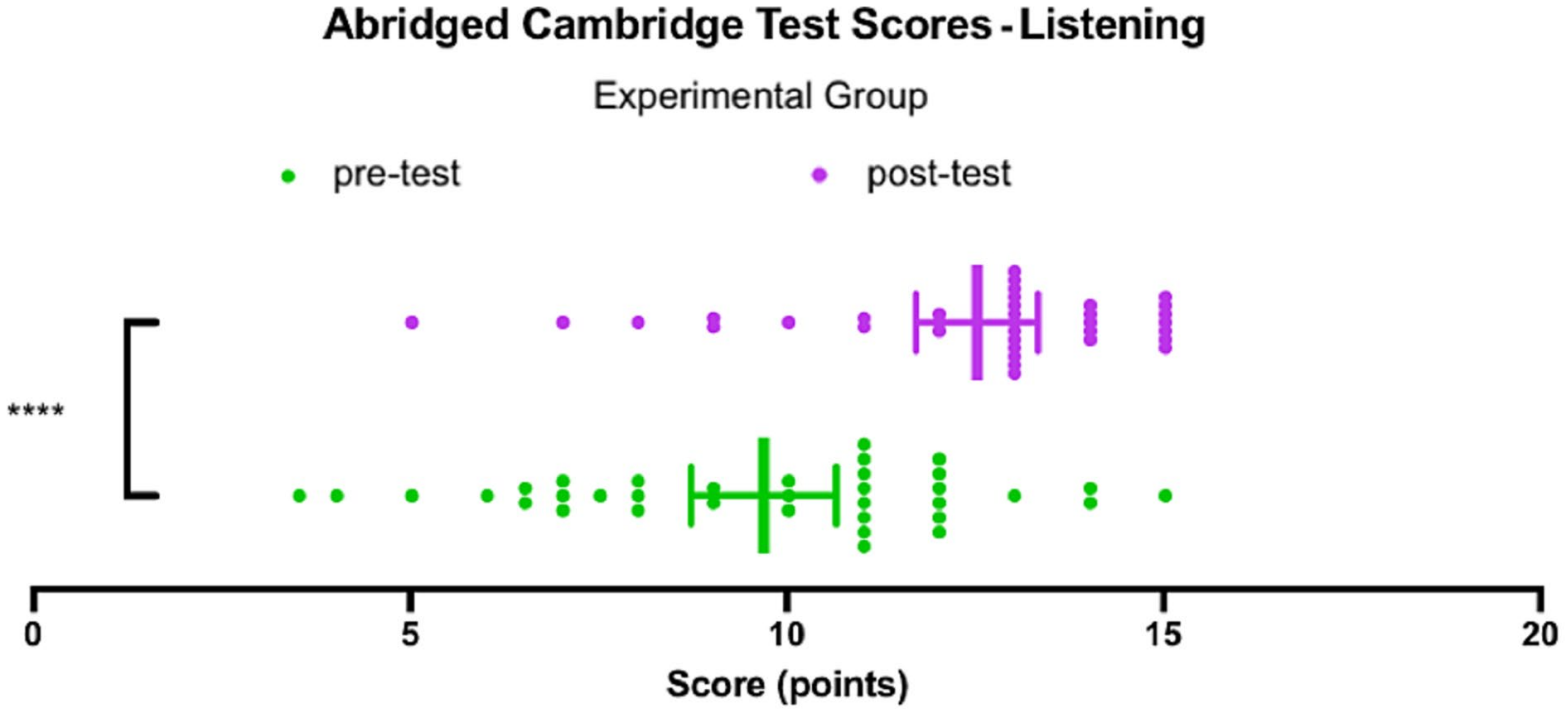
Abridged Cambridge Scores – Listening, Experimental group.

Figure 1 shows the scores achieved by the experimental group on the Listening pre-test and Listening post-test adapted from the *B2 First proficiency exam* designed by the Cambridge Assessment English (2015). On the pre-test, with a full score of 15, the median score is 10.5, and the interquartile range of 4.9 indicates that the middle 50% of the data falls between 12 and 7.1, which corresponds to a rather wide distribution between A1 and lower C1 levels on the official Cambridge English Qualifications scale. The lowest score achieved in the experimental group was 3.5, and the highest score achieved was 15. The mean score of the participants in the experimental group on the pre-test was 9.7 ± 0.5.

On the post-test, the median score is 13, and the interquartile range of 2 indicates that the middle 50% of the data falls between 14 and 12, which is between the low C1 and mid C2 tiers on the official Cambridge English Qualifications scale. The lowest score achieved in the experimental group on the post-test was 5, and the highest score achieved was 15. The mean score of the participants in the experimental group on the post-test was 12.5 ± 0.4.

Since the data passed a normality test, as a group, based on the t-test comparison of the mean scores achieved on the pre-test and the post-test, the experimental group showed a significant improvement of 2.8 ± 0.6 (*p* < 0.0001, *t* = 4.562, df = 70). In other words, after the intervention, the scores achieved by the experimental group increased by a statistically significant margin. The experimental group demonstrated the greatest improvement on the task focused on listening for specific words.

**Figure 2:**
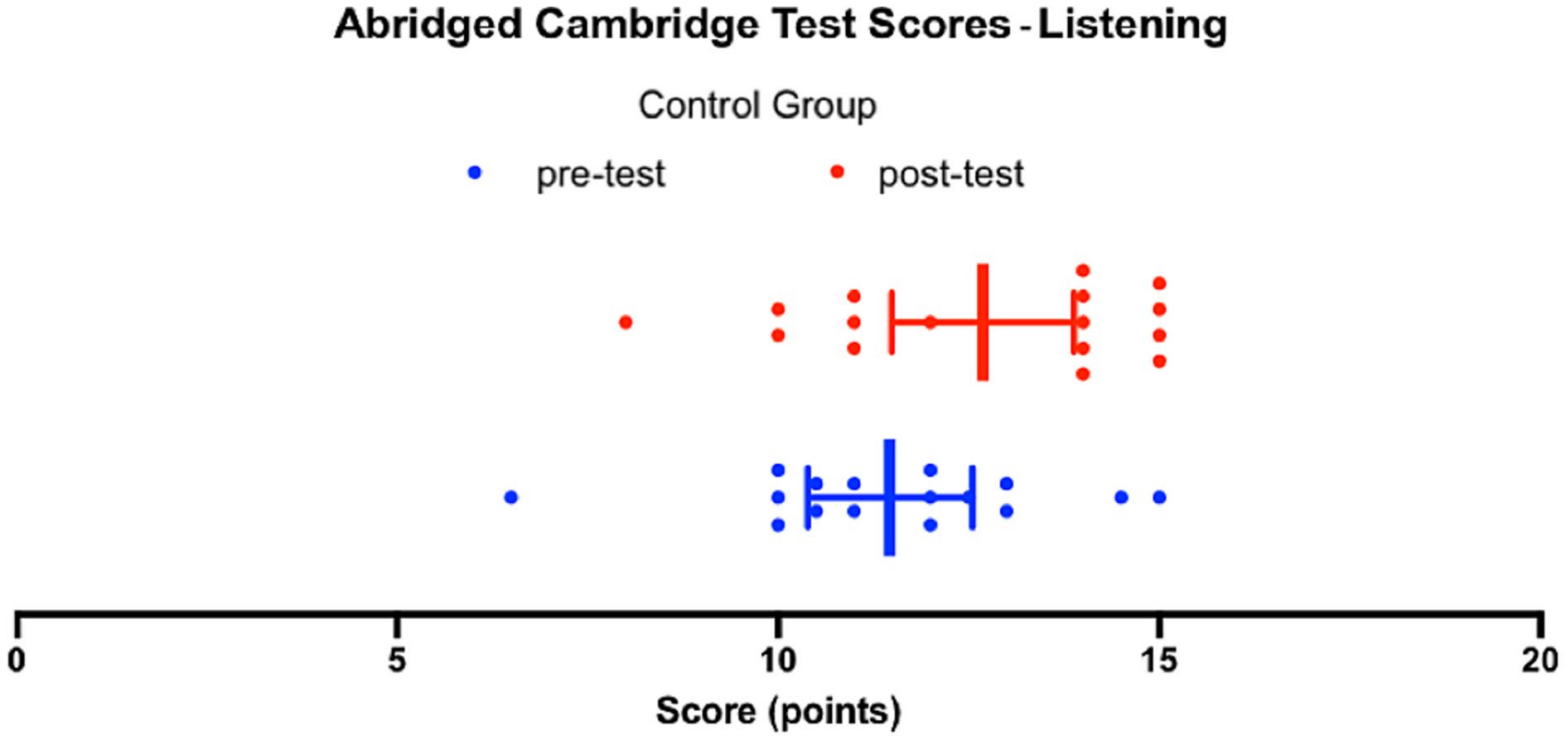
Abridged Cambridge Test Scores – Listening, Control group.

Figure 2 presents the scores achieved by the control group on the listening pre-test and listening post-test adapted from the *B2 First proficiency exam* (Cambridge Assessment English, 2015). On the pre-test, the median score is 11.5, and the interquartile range of 2.8 indicates that the middle 50% of the data falls between 10.1 and 12.9, which corresponds to the upper B2 and upper C1 levels on the official Cambridge English Qualifications scale. The lowest score achieved in the control group was 6.5, while the highest score achieved was 15. The mean score of the students in the control group on the pre-test was 11.5 ± 0.5.

On the post-test, the median score is 14, and the interquartile range of 3.8 indicates that the middle 50% of the data falls between 11.0 and 14.8, which falls between the mid B2 and upper C2 levels on the official Cambridge English Qualifications scale. The lowest score achieved in the control group on the post-test was 8, while the highest score achieved was 15. The mean score of the participants in the control group on the post-test was 12.7 ± 0.6.

Since the data passed a normality test, as a group, based on the t-test comparison of the mean scores achieved on the pre-test and the post-test, the control group showed an improvement of 1.2 ± 0.8 (*p* = 0.1174, *t* = 1.612, df = 30). In other words, after the intervention, the scores achieved by the control group increased, but the difference was not found to be statistically significant.

**Figure 3:**
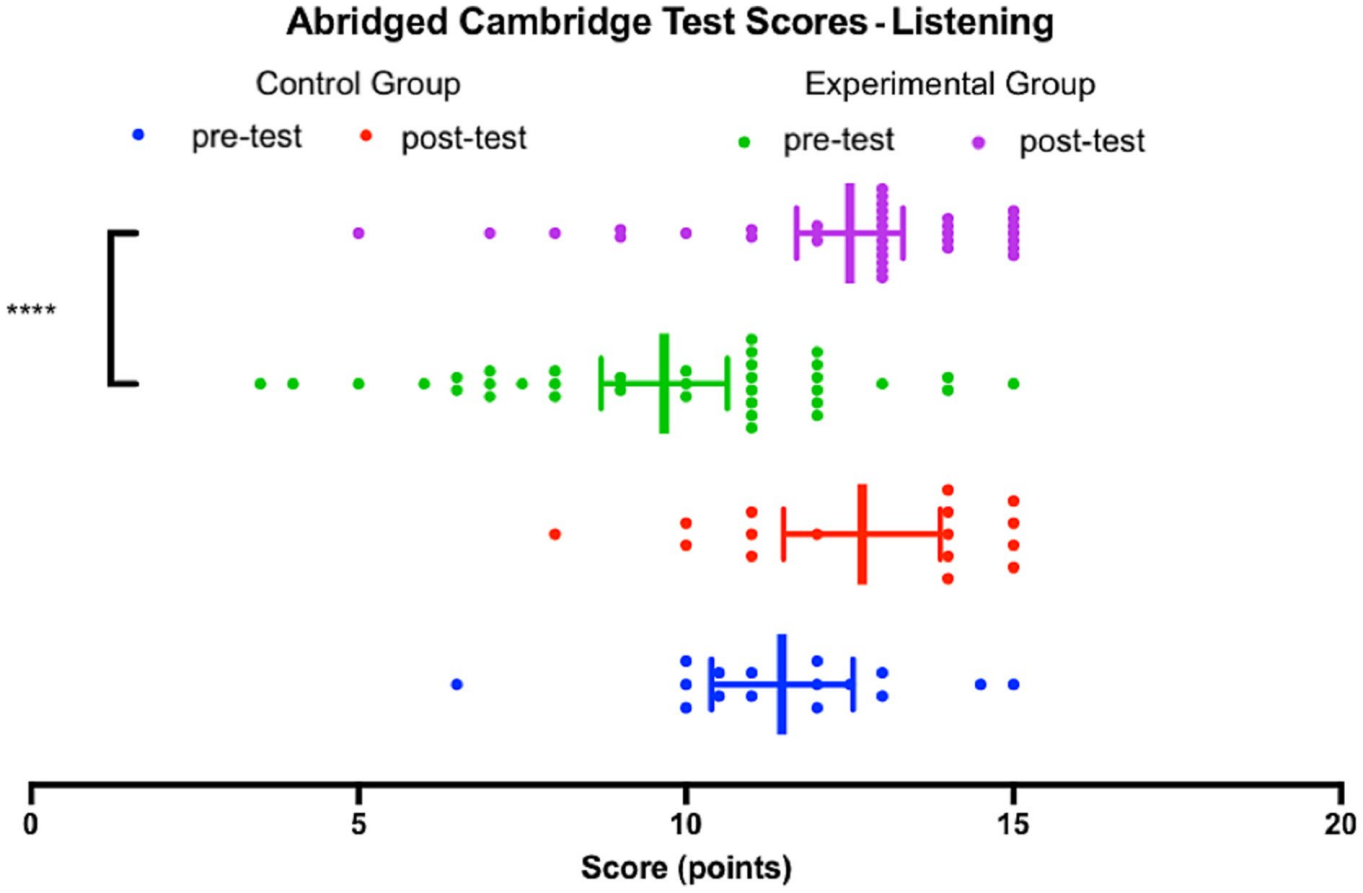
Abridged Cambridge Test Scores – Listening, both groups.

Figure 3 illustrates the comparison of the scores achieved on the Listening pre-test and the Listening post-test by both the experimental group and the control group.

**Figure 4:**
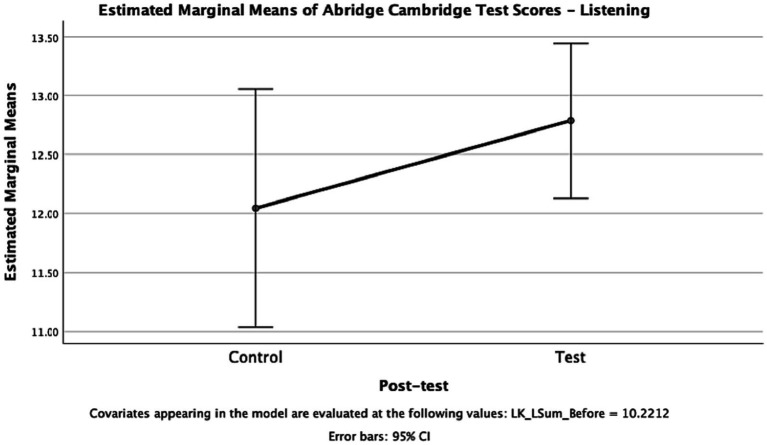
Estimated Marginal Means of Abridged Cambridge Test Scores – Listening.

Figure 4 illustrates the comparison of the estimated means using ANOVA, taking into account the pre-tests as covariates to address potential differences between the control and test groups. The difference in the mean scores on the pre-test was not found to be statistically significant when compared to the control group (*p* = 0.231, *F* = 1.470, eta = 0.231). The estimated marginal means for the two groups, considering the pre-test scores, showed clearly overlapping 95% confidence intervals. Therefore, the difference in the mean scores on the pre-test was not statistically significant, suggesting that both groups had an equal level of proficiency in the English language listening skill for statistical purposes.

Based on the results obtained from the *B2 First proficiency exam* (Cambridge Assessment English, 2015), it can be concluded that only the experimental group, which received the intervention in the form of the flipped classroom teaching model, demonstrated a statistically significant improvement in their listening skills in English. In contrast, the control group, which employed a non-flipped active-learning teaching strategy, did not show a statistically significant effect on the students’ listening skills. These findings suggest that the flipped classroom teaching strategy was more effective in enhancing the participants’ proficiency in listening compared to the non-flipped approach.

**Figure 5:**
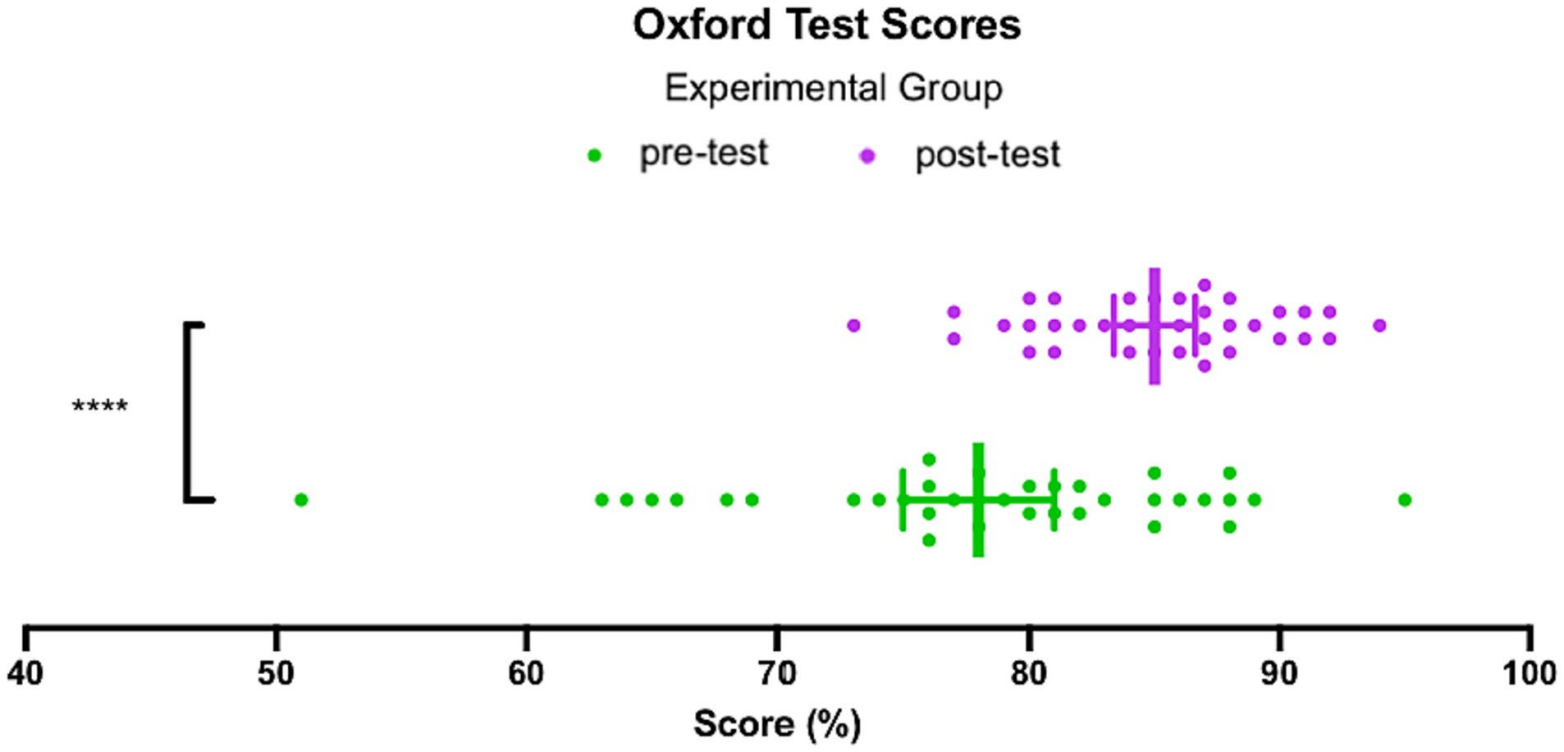
Oxford Test Scores, Experimental group.

Figure 5 presents the scores achieved by the experimental group on the Listening pre-test and Listening post-test, which were adapted from the Listening Test of *Oxford Placement Test 1 and 2* (Allan, 2004b). The exam focused on the participants’ perception and comprehension of pronunciation in the English language. On the pre-test, the median score was 78.5, with an inter-quartile range of 10.7, indicating that the middle 50% of the data ranged from 74.3 to 85.0. According to the official grading rubric of the Oxford Placement Test, these scores correspond to the upper tier of the B2 level. The lowest score achieved in the experimental group was 51, while the highest score achieved was 95. The mean score on the pre-test for the participants in the experimental group was 78.0 ± 1.5.

On the post-test, the median score increased to 85.5, with an inter-quartile range of 7.0, indicating that the middle 50% of the data ranged from 81 to 88. According to the grading rubric, these scores fall within the C1 level. The lowest score achieved in the experimental group on the post-test was 73, while the highest score achieved was 94. The mean score on the post-test for the participants in the experimental group was 85.0 ± 0.8.

Considering that the data passed a normality test, a *t*-test comparison of the mean scores achieved on the pre-test and the post-test was conducted. As a group, the experimental group showed a significant improvement of 7.0 ± 1.7 (*p* < 0.0001, *t* = 4.154, df = 70). In other words, the intervention led to a statistically significant increase in the scores achieved by the experimental group.

### 3.2 Grammatical competence

**Figure 6:**
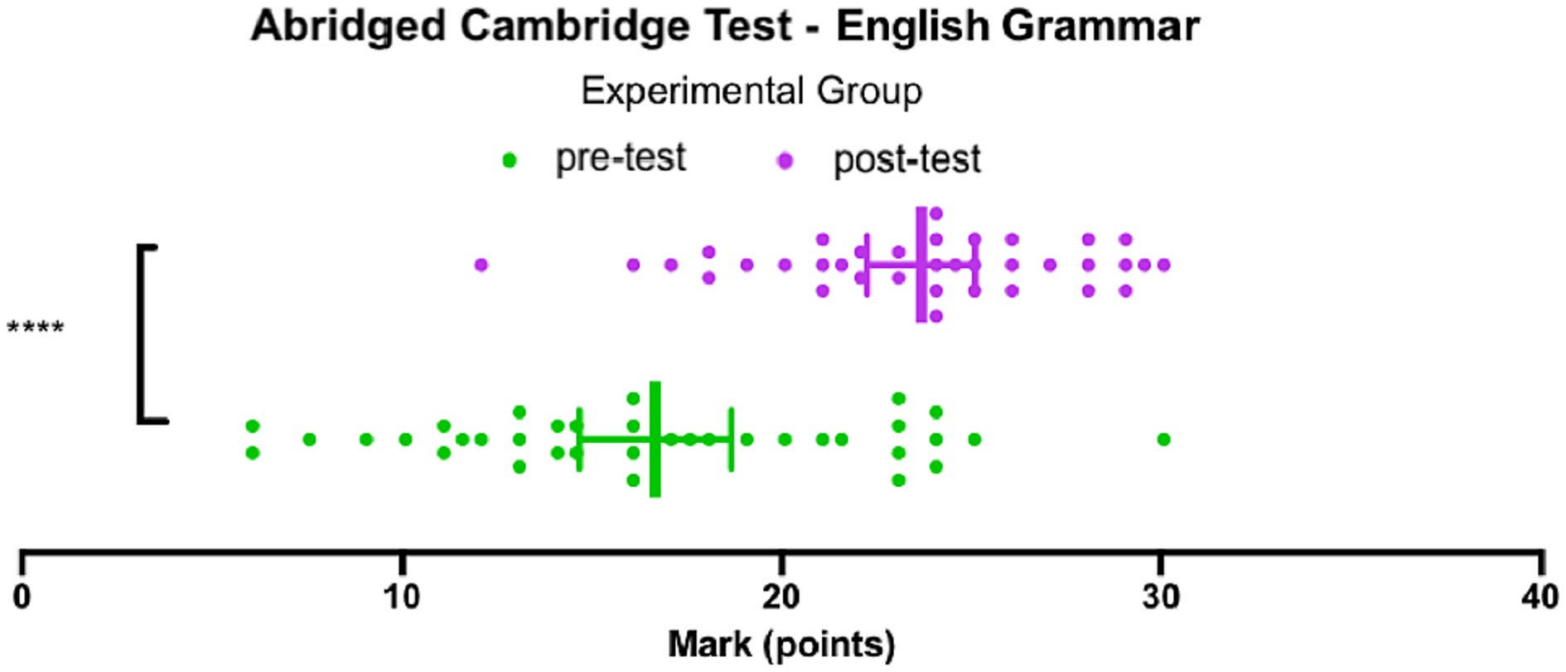
Abridged Cambridge Test – English Grammar, Experimental group.

Figure 6 illustrates the scores achieved by the experimental group on the English Grammar pre-test and English Grammar post-test, which were adapted from the *B2 First proficiency exam* (Cambridge Assessment English, 2015). On the pre-test, with a maximum score of 30, the median score was 16, and the inter-quartile range of 10.4 indicates that the middle 50% of the data ranged from 12.3 to 22.6. According to the official Cambridge English Qualifications, these scores correspond to language levels below A1 and the upper B2 tier. The lowest score achieved in the experimental group was 6, while the highest score achieved was 30. The mean score on the pre-test for the participants in the experimental group was 16.6 ± 1.0.

On the post-test, the median score increased to 24, with an inter-quartile range of 5.75, indicating that the middle 50% of the data ranged from 21 to 26.75. According to the grading rubric, these scores fall between the low B2 and the low C2 tier. The lowest score achieved in the experimental group on the post-test was 12, while the highest score achieved was 30. The mean score on the post-test for the participants in the experimental group was 23.6 ± 0.7.

The data passed a normality test, and based on the t-test comparison of the mean scores achieved on the pre-test and the post-test, the experimental group showed a significant improvement of 7.0 ± 1.2 (*p* < 0.0001, *t* = 5.798, df = 70). In other words, the intervention led to a statistically significant increase in the scores achieved by the experimental group.

**Figure 7:**
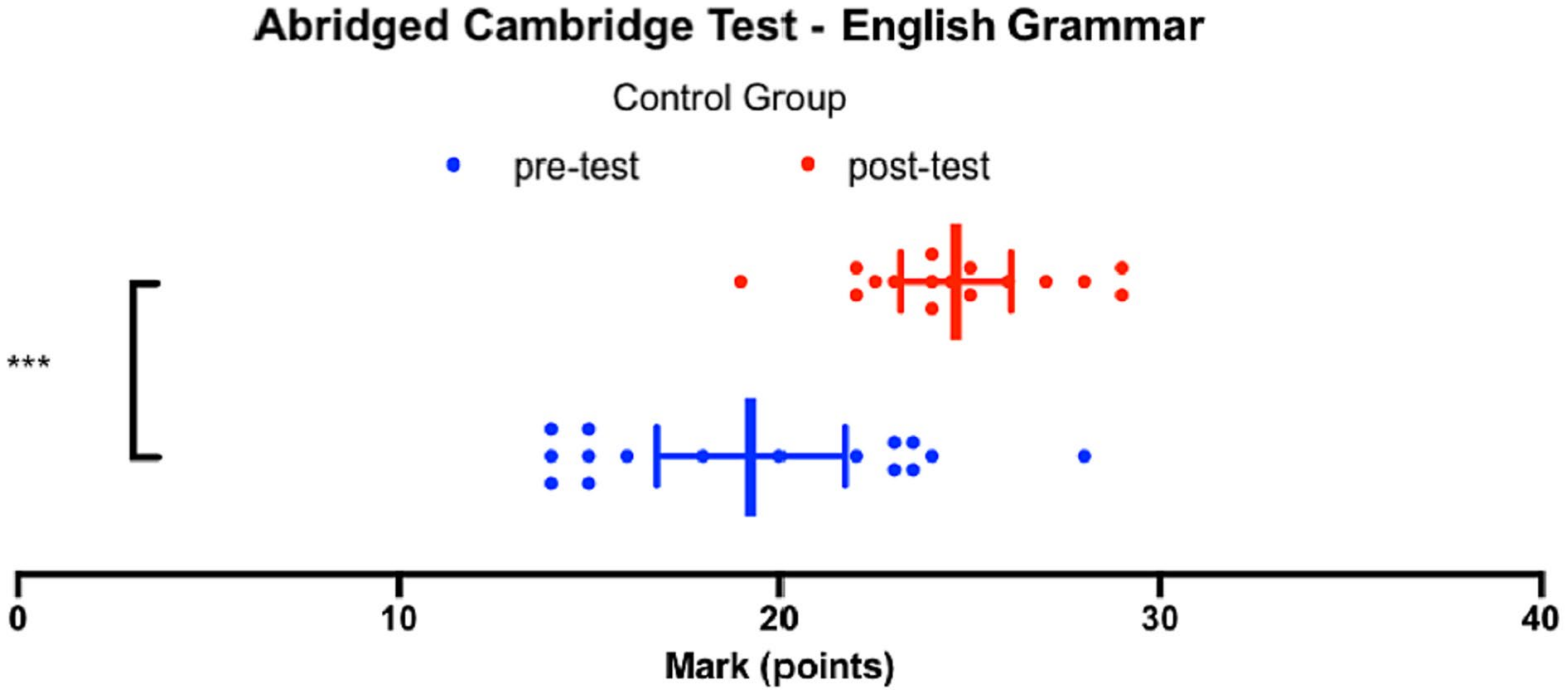
Abridged Cambridge Test – English grammar, Control group.

Figure 7 presents the scores achieved by the control group on the English Grammar pre-test and English Grammar post-test, which were adapted from the *B2 First proficiency exam* (Cambridge Assessment English, 2015). On the pre-test, the median score was 19, and the inter-quartile range of 8.4 indicates that the middle 50% of the data ranged from 15.0 to 23.4. According to the official Cambridge English Qualifications, these scores correspond to the upper A2 and upper B2 tier. The lowest score achieved in the control group was 14, while the highest score achieved was 28. The mean score on the pre-test for the students in the control group was 19.3 ± 1.2.

On the post-test, the median score increased to 24.3, with an inter-quartile range of 4.1, indicating that the middle 50% of the data ranged from 22.6 to 26.8. According to the grading rubric, these scores fall between the upper B2 and lower C2 tier. The lowest score achieved in the control group on the post-test was 19, while the highest score achieved was 29. The mean score on the post-test for the participants in the control group was 24.6 ± 0.7.

The data passed a normality test, and based on the t-test comparison of the mean scores achieved on the pre-test and the post-test, the control group showed a significant improvement of 5.4 ± 1.3 (*p* = 0.0004, *t* = 4.007, df = 30). In other words, the scores achieved by the control group also increased by a statistically significant margin. Both the experimental group, which received intervention in the form of the flipped classroom model of teaching, and the control group demonstrated a statistically significant improvement in their grammar proficiency in English, based on the *B2 First proficiency exam* (Cambridge Assessment English, 2015), by the end of the experiment. Therefore, we can conclude that both the flipped classroom teaching strategy and the active-learning strategy employed in the control group have proven to be effective in teaching English grammar.

**Figure 8:**
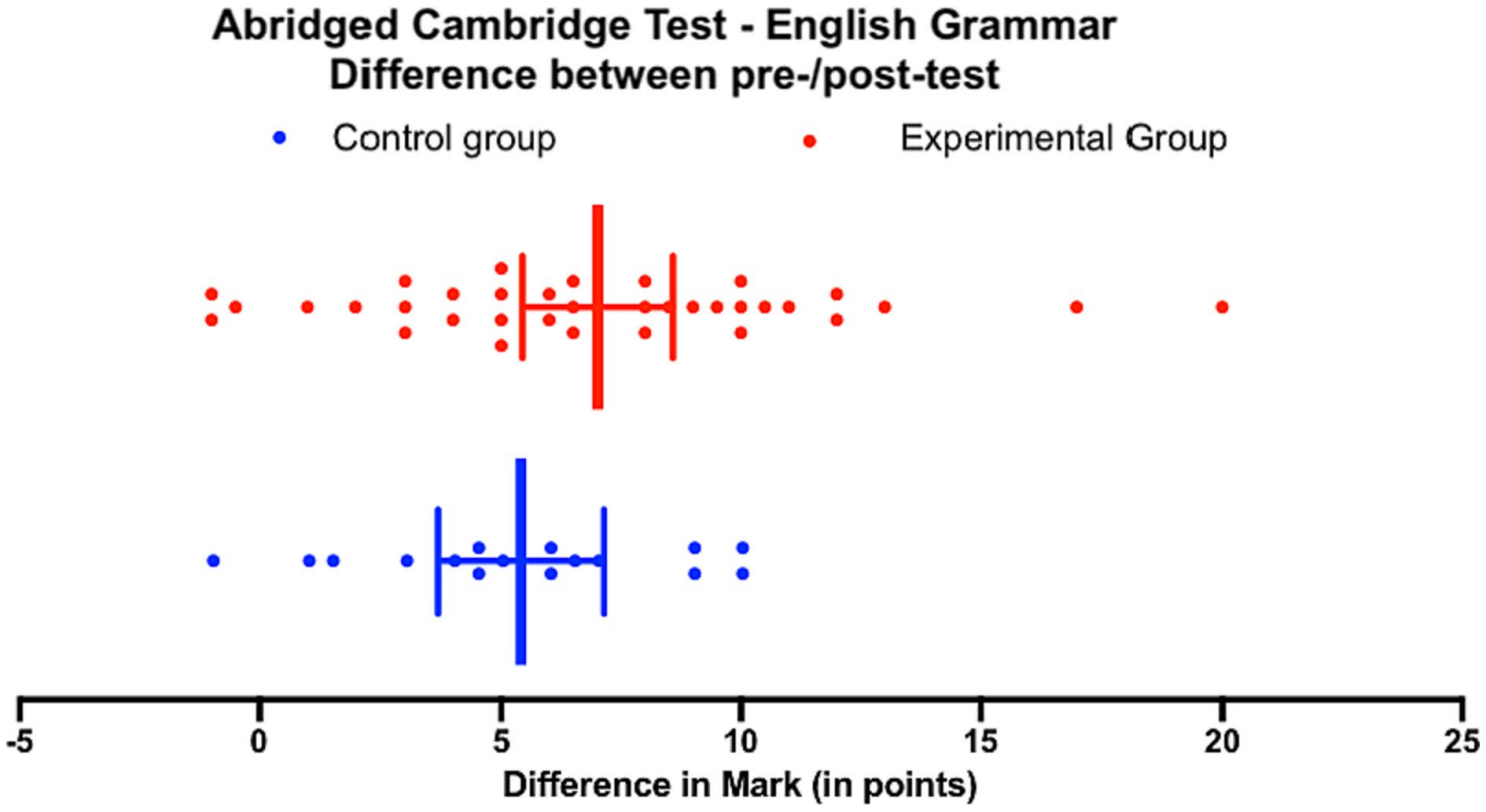
Abridged Cambridge Test – English Grammar, both groups, comparison of difference in scores between pre-test and post-test.

To further investigate the effectiveness of the two teaching strategies, Figure 8 depicts the comparison of improvement between the experimental group and the control group in the English Grammar pre-test and the English Grammar post-test.

**Figure 9:**
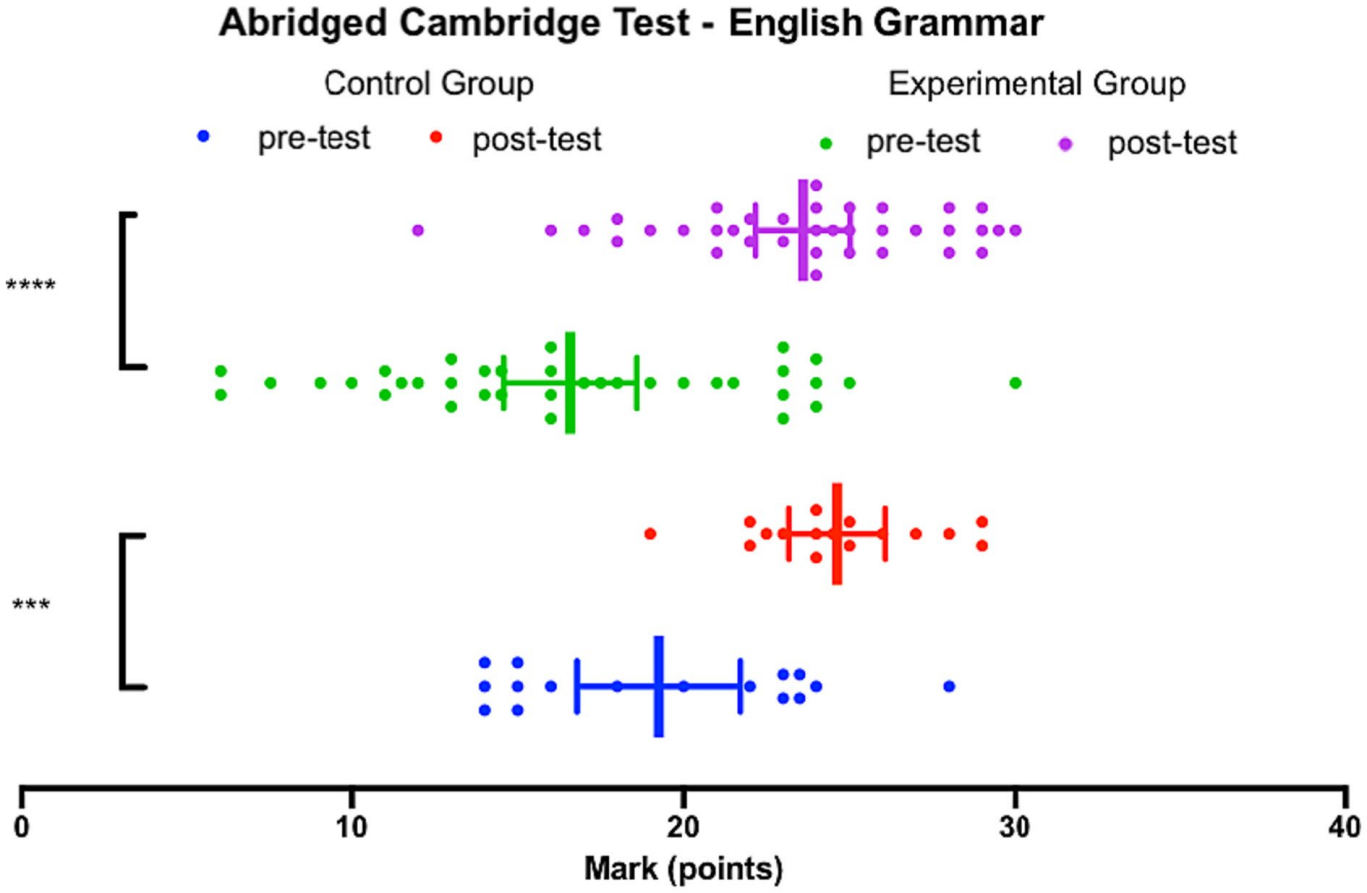
Abridged Cambridge Test – English Grammar, both groups.

Comparing the medians, as the data is not normally distributed, the experimental group showed a median improvement of 6.5 (*n* = 36) as a result of the intervention, while the control group had a median improvement of 5.5 (*n* = 16). Conducting a Mann–Whitney test, it was found that the difference between the two groups was not statistically significant, as indicated by the overlapping 95% confidence intervals in Figure 9 (*p* = 0.2302, *U* = 227).

**Figure 10:**
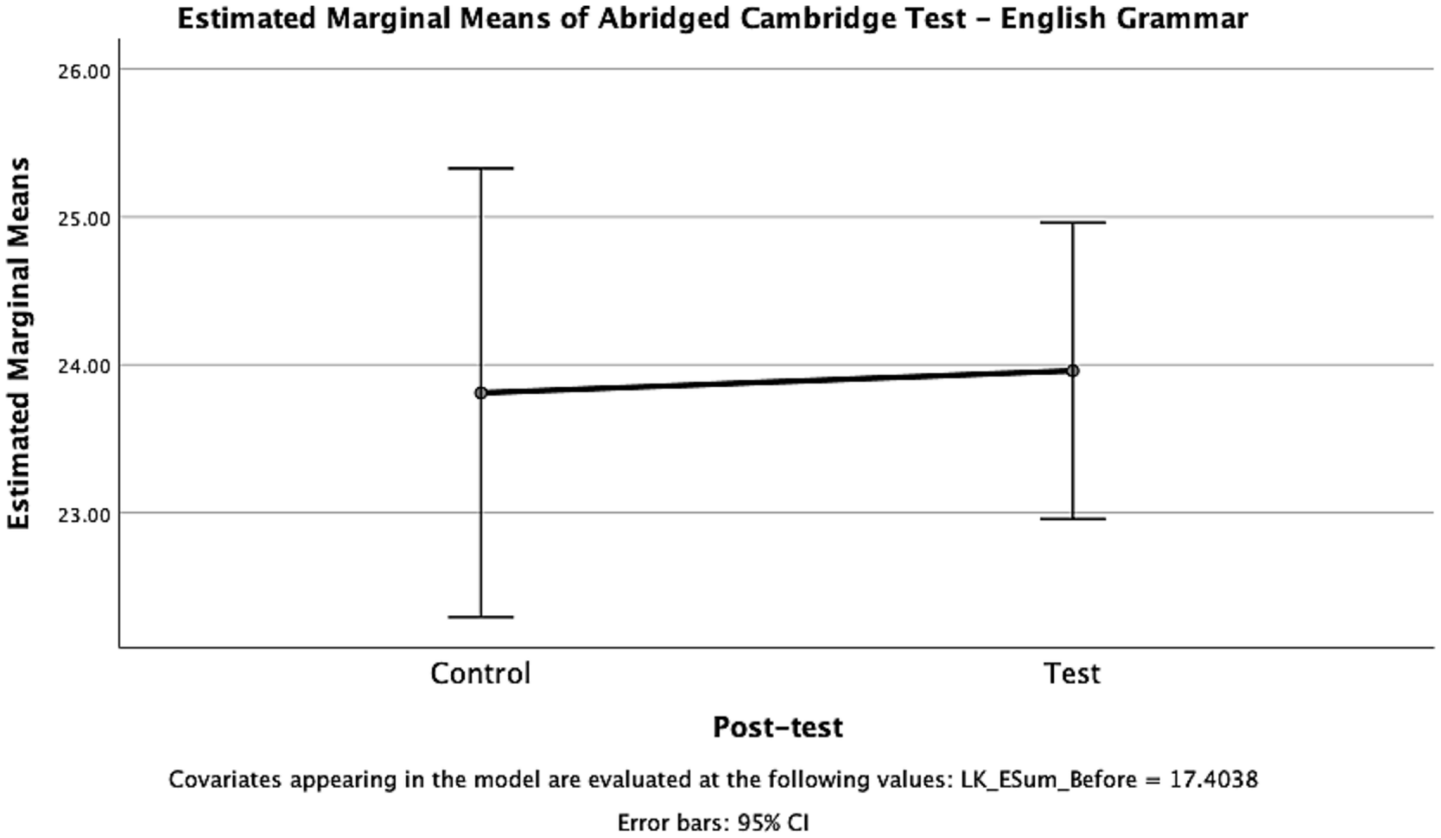
Estimated Marginal Means of Abridged Cambridge Test – English Grammar.

Figure 10 displays the comparison of scores achieved on the English Grammar pre-test and the English Grammar post-test by both the experimental group and the control group, directly using ANCOVA to account for potential differences between the groups by analyzing the pre-tests as covariates. The difference in mean scores on the pre-test was not found to be statistically significant compared to the control group (*p* = 0.871, *F* = 0.027, eta = 0.001). Additionally, Figure 10 presents the estimated marginal means for both groups, considering the pre-test scores, with overlapping 95% confidence intervals. Therefore, it can be concluded that both the experimental group and the control group had an equal level of proficiency in English grammar considering these findings.

Based on the analysis and comparison of the two groups, it can be concluded that there was no statistically significant difference in the improvement on the English grammar post-test between the experimental group and the control group. This suggests that both teaching strategies, the flipped classroom model employed in the experimental group and the active-learning strategy used in the control group, are effective for teaching and learning English grammar. Statistically speaking, neither teaching strategy can be deemed more effective than the other in terms of improving grammar proficiency.

## 4 Discussion

Results of the current research showed improvement in terms of grammar accuracy in both the control and the experimental group. Comparisons with previous studies provide additional insights. Kang’s (2015) study, although conducted under different conditions, found that the flipped classroom intervention had a positive impact on grammatical accuracy. Al-Harbi and Alshumaimeri (2016) reported some improvement in grammar scores but did not find a statistically significant increase. It is worth noting that their study used thematic videos, while the current study used both thematic and grammatical videos. This difference in instructional materials may have contributed to varying results.

Li and Suwanthep (2017), who used pre-class materials focused on grammar similar to the current study, observed significant improvement in general English language proficiency. Al-Naabi and Nizwa (2020) also found a significant improvement in grammar scores in their study. However, both studies faced challenges in ensuring students’ engagement with the pre-class materials.

Obari et al. (2017) combined flipped classroom with blended learning using mobile technologies and found positive outcomes for teaching English, particularly grammar. Lee and Wallace (2018) reported similar results, and Yang (2017) specifically highlighted the suitability of the flipped classroom for teaching English grammar.

Furthermore, Webb and Doman (2016) reported not only a significant improvement in grammar scores but also positive changes in students’ attitudes toward learning grammar.

Flipped classroom has been shown to have a statistically significant effect on students’ listening skills in the English language. This finding was observed in exams assessing listening skills, particularly in the perception of pronunciation item. Roth and Suppasetseree (2016), who focused on the effects of flipped classroom on listening comprehension, also reported positive and statistically significant results. They utilized third-party videos from YouTube, similar to our own method, and their students specifically identified video watching as one of the factors that had the most impact on their listening skills. Similarly, our students also recognized video lectures as one of the greatest advantages of our classes. Another researcher, Ahmad (2016), obtained positive and statistically significant results in listening skills in their experiment with flipped classroom. In this paper, she supports our results in terms that flipped classroom is effective for teaching and training listening comprehension due to increased practice and exposure to authentic language spoken with different accents. Ahmad (2016) further theorizes that the ability for students to pause and rewind video lectures may aid in improving listening comprehension, as it allows them to process spoken language at their own suitable speed, resulting in more meaningful engagement with the language during listening comprehension practice. Unfortunately, she does not provide details on the in-class activities, so it is unclear whether the results were merely a side-effect or if the students received purposeful training in listening skills, similar to our approach in the class. Namaziandost et al. (2019) and Namaziandost et al. (2020) also observed positive effects on English language listening comprehension skills in their students.

## 5 Conclusion

The results of the experiment are positive for research question RQ2 (i.e., Does the suggested model of Flipped Classroom teaching strategy increase learners’ listening skills in the target language more than the non-flipped active-learning strategy used?). The flipped classroom was determined to be significantly more effective than the active-learning strategy in improving the participants’ listening proficiency in the English language. While the flipped classroom showed a significant effect on the results of both listening exams, the active-learning strategy did not have a statistically significant impact on the students’ listening skills. This result came despite the fact that both groups received very similar types of teaching in the classroom and worked on very similar tasks. One of the main reasons for the difference in results may be that the flipped strategy used in the intervention group provided longer exposure to authentic English language. The video-lectures allowed the students to listen to authentic English language, created and spoken by native speakers, exposing them to different accents, pronunciations, paces, intonations, etc. This broadens their English language experience, better preparing them for using the language outside of school. Moreover, the flipped classroom approach provides an element of authentic language immersion, even in a language class where the teacher is not a native speaker. Apart from offering real language experience, the video-lectures also allow the students to observe the speaker, see their lip movements, note their facial expressions and gestures, which are all crucial elements of communication. Additionally, the learner autonomy inherent in the flipped classroom is likely another significant factor contributing to the effects of this teaching strategy. Video-lectures assigned as homework enable students to engage with authentic language content at their own pace, granting them the ability to pause and rewind as needed. This benefits not only shy and slow learners but also curious ones and high-achievers, ultimately leading to more time spent with the target language and improvement in their overall language proficiency (which is the essence of language learning).

For the research questions RQ1 (i.e., Does the suggested model of Flipped Classroom teaching strategy increase the learners’ accuracy in the use of grammar in the target language more than the non-flipped active-learning strategy used?) results are somehow inconclusive. For teaching grammar, both flipped classroom and active-learning were found to have a statistically significant positive effect. Although the flipped classroom showed a greater effect based on the exam results, this difference was not found to be statistically significant. Therefore, both teaching strategies may be considered equally effective for teaching English grammar.

On one hand, this result was somewhat surprising, considering that of the two video-lectures assigned as pre-class tasks before every lecture, one always focused on grammar. It was expected that this would lead to the students’ greater knowledge. On the other hand, the exam was not focused on knowledge; it was centered on the practical skill of using grammar. Had there been a knowledge-oriented exam, perhaps the experimental group would have fared better. However, the primary goal of learning a language is not just to have knowledge of grammar but to be able to use it effectively.

All in all, the implications of this research are high since listening, often referred to as the “Cinderella” of language skills, has frequently been overlooked in EFL classes, leading to students not reaching expected proficiency levels.

One of the main limitations of the study was the inability to establish completely homogenous groups due to group administrative configuration at the University where this research was conducted.

## Data availability statement

The raw data supporting the conclusions of this article will be made available by the authors, without undue reservation.

## Ethics statement

Ethical review and approval was not required for the study on human participants in accordance with the local legislation and institutional requirements. The participants provided their written informed consent to participate in this study.

## Author contributions

LB: Conceptualization, Methodology, Formal analysis, Investigation, Resources, Validation, Writing – original draft, Data curation. RR-C: Conceptualization, Methodology, Supervision, Writing – review & editing. JG-O: Conceptualization, Methodology, Validation, Supervision, Writing – review & editing.
